# LRP5 deficiency down-regulates Wnt signalling and promotes aortic lipid infiltration in hypercholesterolaemic mice

**DOI:** 10.1111/jcmm.12396

**Published:** 2015-02-05

**Authors:** Maria Borrell-Pagès, July Carolina Romero, Lina Badimon

**Affiliations:** a Cardiovascular Research Center, CSIC-ICCC, Hospital de la Santa Creu i Sant Pau, IIB-Sant PauBarcelona, Spain; b Cardiovascular Research Chair, UABBarcelona, Spain

**Keywords:** LRP5, atherosclerosis, plasma cholesterol, canonical Wnt signalling, macrophages

## Abstract

Low-density lipoprotein receptor-related protein 5 (LRP5) is a member of the LDLR family that orchestrates cholesterol homoeostasis. The role of LRP5 and the canonical Wnt pathway in the vascular wall of dyslipidaemic animals remains unknown. In this study, we analysed the role of LRP5 and the Wnt signalling pathway in mice fed a hypercholesterolaemic diet (HC) to trigger dyslipidaemia. We show that *Lrp5*^*−/−*^ mice had larger aortic lipid infiltrations than wild-type mice, indicating a protective role for LRP5 in the vascular wall. Three members of the LDLR family, *Lrp1*, *Vldlr* and *Lrp6*, showed up-regulated gene expression levels in aortas of *Lrp5*^*−/−*^ mice fed a hypercholesterolaemic diet. HC feeding in *Lrp5*^*−/−*^ mice induced higher macrophage infiltration in the aortas and accumulation of inflammatory cytokines in blood. Wnt/β-CATENIN signalling proteins were down-regulated in HC *Lrp5*^*−/−*^ mice indicating that LRP5 regulates the activation of Wnt signalling in the vascular wall. In conclusion, our findings show that LRP5 and the canonical Wnt pathway down-regulation regulate the dyslipidaemic profile by promoting lipid and macrophage retention in the vessel wall and increasing leucocyte-driven systemic inflammation.

## Introduction

Hypercholesterolaemia is a causal factor for atherosclerosis, which predisposes individuals to the development of clinical cardiovascular diseases 1. High levels of low-density lipoprotein (LDL) in plasma rapidly infiltrate the vessel wall triggering an inflammatory–immunomodulatory chain reaction 2,3. Members of the LDL receptor family are involved in lipoprotein transport and plasma LDL cholesterol clearance, modulating critical stages of atherosclerosis progression including inflammation, foam cell formation and endothelial activation 4,5. There is ongoing controversy on the role of LRP5 and the canonical Wnt/β-CATENIN pathway in lipid induced vascular damage and atherosclerosis. Indeed, activation of the β-CATENIN/lymphoid enhancer-binding factor 1 (LEF1) signalling in the endothelium occurs in response to atheroprone haemodynamic stimulations and precedes lesion development in *ApoE*^*−/−*^ mice 6 and higher levels of active β-CATENIN are observed in disrupted atherosclerotic plaques compared to stable plaques from human carotid artery, suggesting a potential role for Wnt signalling in the evolution of atherosclerotic plaque 7. However, *ApoE*^*−/−*^*Lrp5*^*−/−*^ mice fed a high-fat diet developed bigger atherosclerotic lesions that their *ApoE*^*−/−*^ littermates 8, although the exceedingly high levels of cholesterol in these animals (almost 750 mg/dl) could have shadowed any effect of the canonical Wnt receptor, LRP5.

The canonical Wnt/β-CATENIN pathway has also been described to regulate inflammatory reactions although its role remains controversial. Indeed, Wnt/β-CATENIN pathway seems to inhibit inflammation as β-CATENIN inhibitors increase the expression of inflammatory genes in human aortic endothelial cells 9 and administration of GSK3β, an inhibitor of Wnt/β-CATENIN pathway in human monocytes triggers Toll-like receptor-mediated pro-inflammatory cytokine production 10. However, IL-1β and LPS induced nuclear β-CATENIN accumulation in human vascular endothelial cells 6 and activation of canonical Wnt genes have been found in endothelial cells of a rejected kidney model 11 suggesting that activation of the pathway triggers the inflammatory response.

We have recently reported that LRP5 is involved in monocyte to macrophage differentiation 12, it regulates macrophage motility and LRP5-expressing mononuclear cells are a fraction of the macrophages found in human advanced coronary atherosclerotic plaques 13. Still, the presence of LRP5-positive cells in these coronary plaques does not imply causality. Thus, to better understand the role of LRP5 and Wnt signalling in the early stages of lipid infiltration in the vessel wall, we studied the effects of a hyperlipidaemic diet inducing a mild increase in cholesterol serum levels in *Lrp5*^*−/−*^ mice and in wild-type (WT) controls. We hypothesized that LRP5 and the Wnt signalling pathway have a role in the inflammatory process associated to atherosclerosis progression. Absence of LRP5, induced higher lipid infiltration in mouse thoracic aortas, increased the transcription of the LDLR family member *Lrp6*, induced higher macrophage infiltration and increased inflammatory cytokines secretion, supporting an anti-inflammatory role for LRP5 and the target genes of the Wnt/β-CATENIN pathway.

## Materials and methods

### Animals and experimental design

*Lrp5*^*−/−*^ mice, a kind gift from Dr. Bart Williams 14–16 were maintained in a C57BL/6 background. Mice were housed in cages under controlled temperature (21 ± 2°C) on a 12 hrs light/dark cycle with food and water *ad libitum*. Homozygous WT *C57BL/6* mice (*n* = 22) and *Lrp5*^*−/−*^ *C57BL/6* mice (LRP5^*−*/*−*^; *n* = 22) were used for the protocols. The presence of *Lrp5* alleles was assessed by PCR amplification from DNA extracted from tail biopsies in WT, heterozygous and homozygous littermates. Primers used were S17 (GGC TCG GAG GAC AGA CCT GAG), S23 (CTG TCA GTG CCT GTA TCT GTC C) and IRES31 (AGG GGC GGA ATT CGA TAG CT). *Lrp5*^*−/−*^ and *Wt* mice were fed a normal chow diet (NC, Tekland diet, Harland Labs Berkeley, CA, USA) for 10 weeks. Animals were then divided into two groups to be fed NC or high cholesterol diet (HC, TD.88137, Harland Labs) for further 8 weeks (8–12 mice/group). Cardiac puncture was performed in mice under terminal anaesthesia (1 mg/kg Medetomidine and 75 mg/kg Ketamine, ip). The study protocol was conducted in conformity with the Public Health Service (PHS) Policy on Humane Care and Use of Laboratory Animals and approved by the local institutional animal research committee (ICCC051/5422).

### Biochemical analysis and blood-derived mRNA

Blood samples were collected in serum separator gel tubes and PAX-tubes. Serum was obtained by centrifugation 1200g 20 min. at 4°C. Cholesterol, triglycerides and HDL levels were measured enzymatically by using commercially available kits (GERNON reagents) and read in a spectrophotometer (MC-15 SOFT; RAL). PAX-tubes were processed for preparation of blood-derived mRNA using PAXgene Blood RNA Kit (Qiagen Inc, Valencia, CA, USA). Real Time RT-PCR array was performed with RT2 Profiler PCR array PAMM-021 (SABiosciences, Qiagen).

### Quantification of atherosclerotic lesions

Mice were anaesthetized and aortas were removed, carefully cleaned of adventitial fat under a stereoscopic microscope, and longitudinally cut with the luminal surface facing up (*n* = 6–8 mice/group). Aortas were fixed overnight in 4% paraformaldehyde, washed with ddH_2_O 1 hr in gentle shaking and stained with Oil-red-O (ORO) for 30 min. Aortas were rinsed with 70% ethanol and ddH_2_O; images were captured by Nikon Instruments, Melville, NY, USA Eclipse 80i microscope and digitized by Retiga 1300i QImaging, Surrey, BC, Canada Fast camera. ORO-stained area was quantified with Image J software and results are expressed as percentage of lipid area/total aortic area.

### Thin layer chromatography

Aorta tissue (5 mg) was homogenized in NaOH 0.1 M. The organic solvent was removed under a N_2_ stream, the lipid extract was suspended in dichloromethane and separated by thin layer chromatography (TLC). TLC was performed on silica G-24 plates. Concentrations of standards (a mix of cholesterol, cholesterol palmitate, triglycerides, diglycerides and monoglycerides) were applied to each plate. The chromatographic developing solution was heptane/diethylether/acetic acid (74:21:4, vol/vol/vol). The spots corresponding to cholesteryl esters (CE) were quantified by densitometry.

### Real time RT-PCR

Aortas were frozen in liquid nitrogen and aortic RNA was isolated with Trizol® Reagent (Invitrogen; Carlsbad, CA, USA *n* = 5–7 mice/group). Concentration was determined with a NanoDrop ND-1000 spectrophotometer (NanoDrop Technologies, Inc., Wilmington, DE, USA) and purity was checked by the A260/A280 ratio (ratios between 1.8 and 2.1 were considered acceptable). cDNA was synthesized from 0.5 μg RNA with cDNA Reverse transcription kit (Qiagen). The resulting cDNA samples were amplified with a RT-PCR thermal cycler (Applied Biosystems Carlsbad, CA, USA 7900HT) and the following specific probes from CONDA: *Lrp5* (Mm.PT.49a.8045420), *Lrp1* (Mm.PT.49a.7750137), *Lrp2* (Mm.PT.49a.11916154), *Lrp6* (Mm.PT.56a.6383636), *Lrp8* (Mm.PT.49a.6553055), *Ldlr* (Mm.PT.49a.9930556) and *Cd36* (Mm.PT.49a.12111555). *Vldlr* (Mm00443298_m1) was purchased from Applied Biosystems. Results were normalized with *18S* probe from Applied Biosystems.

### Immunohistochemistry

Immediately after surgical excision, aortas were immersed in fixative solution (4% paraformaldehyde) and embedded in paraffin, cut into 5 μm thick serial sections and placed on poly-L-lysine coated slides. Primary antibodies used were: Matrix Metalloproteinase-7, MMP-7 (Rabbit polyclonal; Abcam), Cambridge, UK β-CATENIN (Rabbit policlonal; Millipore) Bedford, MA, USA and HAM56 (Mouse monoclonal; Dako Glostrup, Denmark). Before incubation with primary antibodies, sections were washed and endogenous peroxidase activity was impressed with H_2_O_2_ and goat or horse serum block. Primary antibodies were detected using the avidin–biotin immunoperoxidase technique. Sections were incubated with an appropriate biotinylated secondary antibody (1:200; Vector Laboratories). Burlingame, CA, USA The chromogen used was 3,3′-diaminobenzidine. Haematoxylin was used for nuclear stain. Images were captured by Nikon Eclipse 80i microscope and digitized by Retiga 1300i Fast camera, magnification (×400).

### Statistical analysis

Results are expressed as mean ± SEM. A Stat View statistical package was used for all the analysis. Comparisons among groups were performed by two-way anova analysis. Regression analyses were performed by applying *Y* = *a* + b*X lineal pattern selecting just highly adjusted equations. Slopes were compared by *t*-test. Statistical significance was considered when *P* < 0.05.

## Results

### Serum cholesterol profile

**Fig 1:**
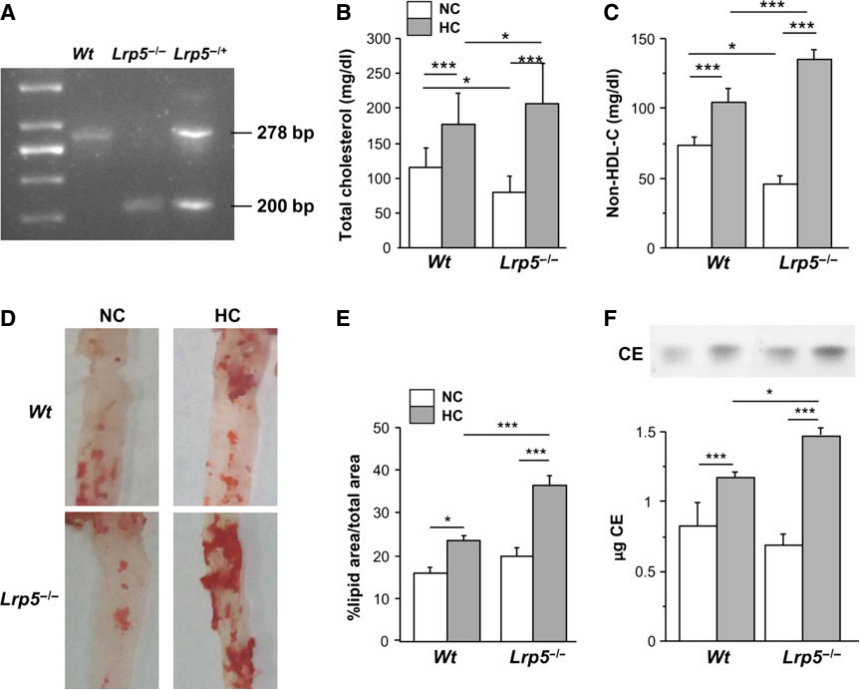
Hypercholesterolaemic (HC) *Lrp5*^*−/−*^ mice model characterization. (A) Agarose gel showing *Wt*, *Lrp5*^*−/−*^ and *Lrp5*^*−/+*^ alleles. (B) Serum cholesterol levels in *Wt* and *Lrp5*^*−/−*^ mice fed a normocholesterolaemic (NC) or a HC diet. (C) Non-HDL-C in *Wt* and *Lrp5*^*−/−*^ mice fed a NC or a HC diet. (D) Representative images of mouse thoracic aortas stained with ORO. (E) Quantification of lipid area in mice aortas. (F) Cholesteryl ester measurement by thin layer chromatography and bar graph showing CE quantification. *P < 0.05; ***P < 0.005.

We analysed the differences in serum cholesterol profiles of *Wt* and *Lrp5*^*−/−*^ mice fed a normocholesterolaemic (NC) or hypercholesterolaemic (HC) diet. Figure1A shows an agarose gel with *Wt, Lrp5*^*−/−*^ and *Lrp5*^*+/−*^ alleles. Cholesterol serum levels of *Lrp5*^*−/−*^ mice fed a NC diet containing 3.5% (w/w) fat and 0% cholesterol were lower than those of their *Wt* littermates (Fig.1B). When mice were fed a HC diet containing 21% (w/w) fat and 0.25% cholesterol, total cholesterol levels were significantly increased in both *Lrp5*^*−/−*^ and *Wt* mice (Fig.1B). Interestingly, the overall increase in serum cholesterol levels in high-fat diet with respect to chow diet was double in *Lrp5*^*−/−*^ (125.69 mg/dl) than in *Wt* mice (62.25 mg/dl, Fig.1B). Non-HDL cholesterol increased by 42 ± 8% and 300 ± 23% in HC *Wt* and HC *Lrp5*^*−/−*^ mice with respect to their NC littermates (Fig.1C).

### Lipid deposition in aortas

The administration of a HC diet in *Wt* and *Lrp5*^*−/−*^ mice led to a significant increase in early lesions in the aortic area with lipid infiltration in both groups. Indeed, the aortic lipid rich coverage increased by 7 ± 0.5% and by 17 ± 1% in *Wt* and *Lrp5*^*−/−*^ mice respectively (Fig.1D and E). The increased lipid deposition in HC *Lrp5*^*−/−*^ mice was over 70% higher than in HC *Wt* mice indicating a protective role for LRP5 in mice aortas. Furthermore, HC feeding induced higher levels of CE accumulation in *Lrp5*^*−/−*^ (87 ± 8%) than in *Wt* (51 ± 6%) aortas with respect to NC mice (Fig.1F).

### Positive correlation between plasma cholesterol levels and aortic lipid deposition in Wt and Lrp5^*−*/*−*^ mice

**Fig 2:**
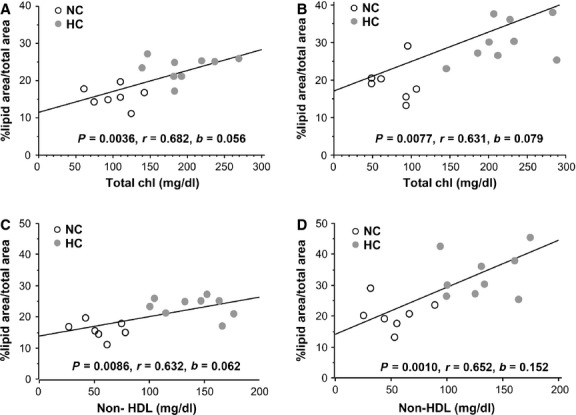
Regression analyses between total cholesterol and aortic lipid coverage in *Wt* (A) and *Lrp5*^*−/−*^ mice (B) and between non-HDL cholesterol and aortic lipid coverage in *Wt* (C) and *Lrp5*^*−/−*^ mice (D) with their statistical significances (p), correlation coefficients (r) and slopes (b).

To analyse the effect of total plasma cholesterol on aortic lipid coverage, regression analyses were performed for *Wt* and *Lrp5*^*−/−*^ mice results. Significantly higher aortic lipid deposition was observed with higher plasma cholesterol levels (Fig.2A and B). The slope for this correlation was steeper for *Lrp5*^*−/−*^ mice. Correlation analyses remained positive and significant between non-HDL cholesterol and aortic lipid coverage and slopes were significantly different with higher change in *Lrp5*^*−/−*^ mice (Fig.2C and D).

### Increased macrophage infiltration in Lrp5^*−*/*−*^ mice

**Fig 3:**
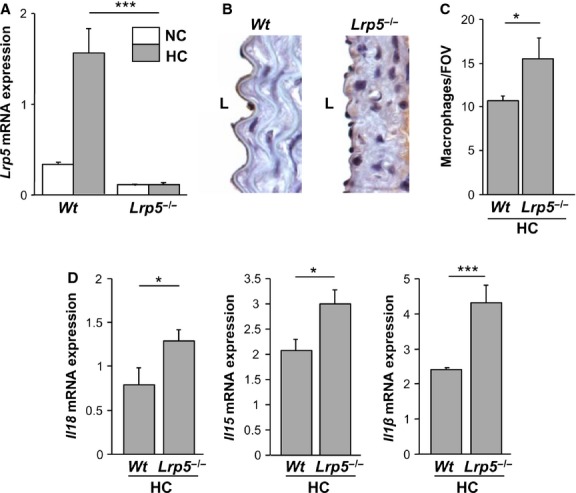
Hypercholesterolaemic (HC) *Lrp5*^*−/−*^ mice show increased aortic macrophage infiltration. (A) *Lrp5* expression levels in blood leucocytes in normocholesterolaemic (NC) or HC *Wt* and *Lrp5*^*−/−*^ mice. (B) HC *Wt* and HC *Lrp5*^*−/−*^ mice aortas labelled with HAM56, L, lumen. (C) Quantitative analysis of B expressed as number of macrophages/field of vision. (D) White blood cells gene expression of pro-inflammatory cytokines in HC *Wt* and HC *Lrp5*^*−/−*^ mice. *P < 0.05; ***P < 0.005.

*Lrp5* mRNA expression in white blood cells showed a 77.5 ± 0.2% increase because of hypercholesterolaemia in *Wt* mice (Fig.3A). *Lrp5* mRNA expression was negligible in white cells of *Lrp5*^*−/−*^ mice. Immunostaining with HAM56 for macrophages (Fig.3B) showed a 47.6 ± 2% increase in macrophage infiltration to the intima in HC *Lrp5*^*−/−*^ mice compared with HC *Wt* mice (Fig.3C). Monocytes–macrophages in aortic tissue were more abundant in *Lrp5*^*−/−*^ mice.

### Expression of pro-inflammatory cytokines in white blood cells

We then analysed gene expression levels of pro-inflammatory cytokines in white blood cells from HC *Wt* and HC *Lrp5*^*−/−*^ mice. Results show an increase of *Il18*, *Il15* and *Il1*β mRNA expression by 62.5 ± 2%, 43 ± 1% and 91 ± 2% respectively in HC *Lrp5*^*−/−*^ compared with HC *Wt* mice (Fig.3D). These results show increased inflammation in HC *Lrp5*^*−/−*^ mice respect to their *Wt* littermates.

### Wnt pathway activation in mice aortas

**Fig 4:**
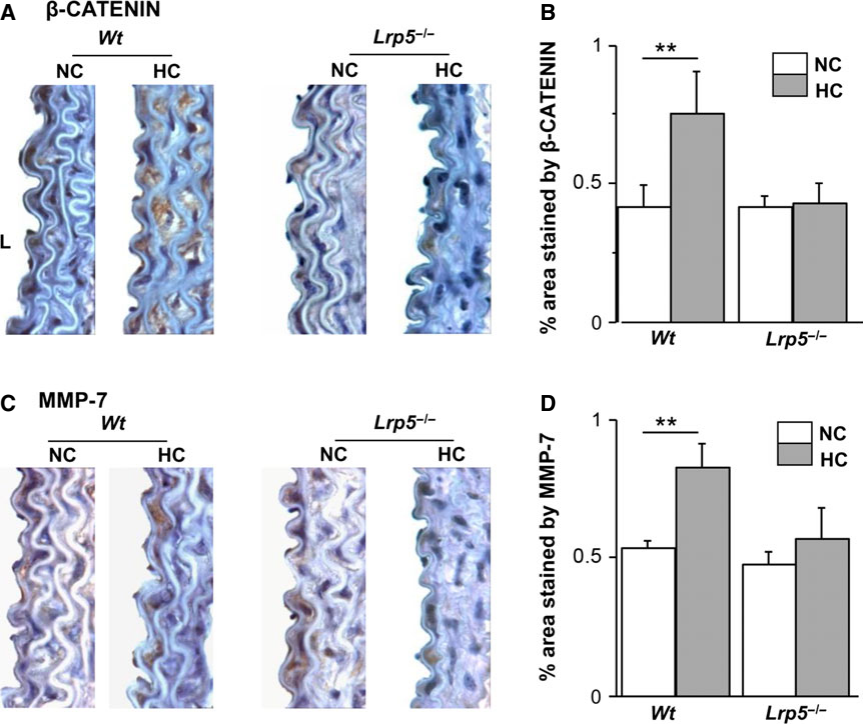
Wnt pathway modulation in aortas from *Wt* and *Lrp5*^*−/−*^ mice. (A) Representative images of aortas sections immunostained with β-CATENIN in normocholesterolaemic (NC) or hypercholesterolaemic (HC) *Wt* and *Lrp5*^*−/−*^ mice. (B) % area stained by β-CATENIN. (C) Representative images of aortas sections immunostained with MMP-7 in NC or a HC *Wt* and *Lrp5*^*−/−*^ mice. (D) Quantitative analysis of aortas in C. **P < 0.01.

Levels of two proteins of the canonical Wnt pathway, β-CATENIN and MMP-7 were analysed in macroscopically ORO-stained areas of the aortas of *Wt* and *Lrp5*^*−/−*^ mice. β-CATENIN staining was increased in aortas of HC *Wt* mice compared to NC *Wt* mice indicating an activation of the Wnt/β-CATENIN pathway by hypercholesterolaemia. As expected, there was no increase in β-CATENIN staining in HC *Lrp5*^*−/−*^ mice (Fig.4A and B). Similarly MMP-7, another downstream protein of the pathway, was up-regulated in HC *Wt* compared with NC *Wt* mice, while in aortas from HC *Lrp5*^*−/−*^ mice, the area covered by MMP-7 staining was smaller (Fig.4C and D). These results suggest that the canonical Wnt/β-CATENIN pathway is triggered by mild dyslipidaemia as a protective response and that this effect cannot be produced when LRP5 is absent.

### LDL receptors in WT and LRP5^*−*/*−*^ mice aortas

**Fig 5:**
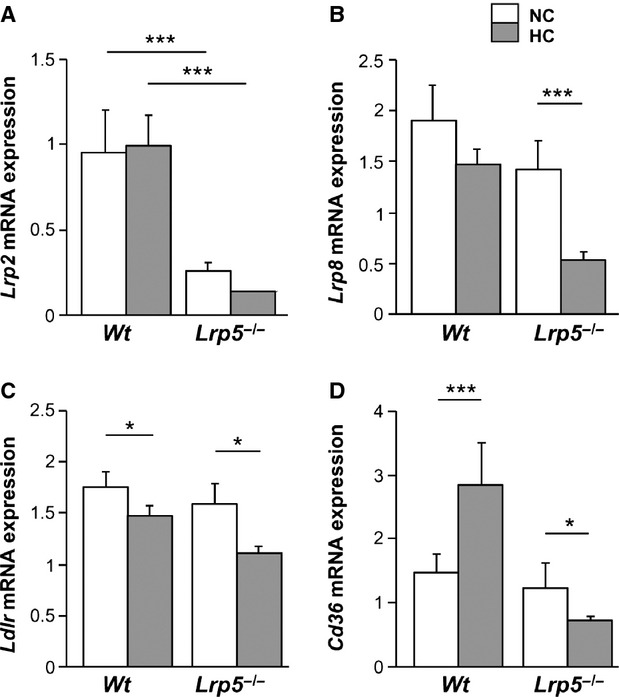
Receptors expression in mice aortas. mRNA expression levels of *Lrp2* (A), *Lrp8* (B), *Ldlr* (C) and *Cd36* (D) in aortas from *Wt* and *Lrp5*^*−/−*^ mice fed a normocholesterolaemic (NC) or a hypercholesterolaemic (HC) diet. *P < 0.05; ***P < 0.005.

**Fig 6:**
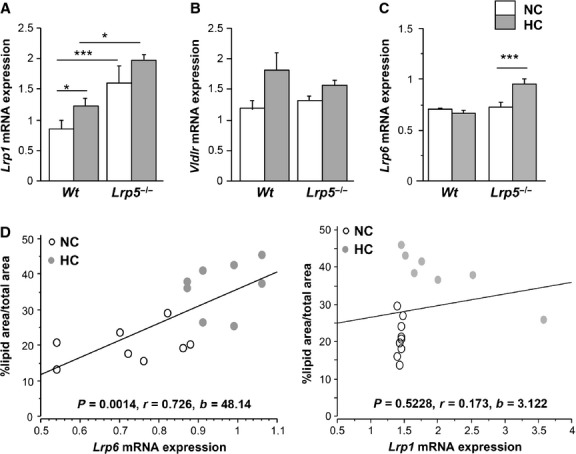
Receptors expression in mice aortas. mRNA expression levels of *Lrp1* (A), *Vldlr* (B) and *Lrp6* (C) in aortas from *Wt* and *Lrp5*^*−/−*^ mice fed a normocholesterolaemic (NC) or a hypercholesterolaemic (HC) diet. *P < 0.05; ***P < 0.005. (D) Regression analyses between aortic lipid coverage and *Lrp6* or *Lrp1* gene expression levels in aortas of *Lrp5*^*−/−*^ mice with their statistical significances (p), correlation coefficients (r) and slopes (b).

LRP5 belongs to the LDL receptor superfamily of proteins involved in lipoprotein trafficking 5. To determine if other receptors were causing the lipid infiltration observed in aortas of *Lrp5*^*−/−*^ mice, we analysed gene expression of receptors described to be involved in the initial stages of atherosclerosis lesions. Results show that *Lrp2* gene expression was strongly down-regulated in *Lrp5*^*−/−*^ mice independently of diet (Fig.5A), while *Lrp8* gene expression was down-regulated in *Wt* and *Lrp5*^*−/−*^ mice after HC diet (21.05 ± 2% and 57.14 ± 2% respectively, Fig.5B). Similarly, the classical *Ldlr* mRNA expression levels were reduced with HC feeding in both genotypes (21.4 ± 0.5% in HC *Wt* respect to NC *Wt* and 23.6 ± 1% in *Lrp5*^*−/−*^, Fig.5C). *Cd36* increased its mRNA expression by 48.2 ± 1% in HC *Wt* mice with respect to NC *Wt* animals, but showed a 41.6 ± 0.5% decrease in HC *Lrp5*^*−/−*^ mice with respect to NC *Lrp5*^*−/−*^ mice (Fig.5D). On the contrary, *Lrp1* significantly increased by 37 ± 2% in HC *Wt* mice and 21 ± 9% in HC *Lrp5*^*−/−*^ mice aortas with respect to their NC littermates, but there was no significant effect because of HC in the *Lrp5*^*−/−*^ mice (Fig.6A). The *Vldlr* mRNA levels were up-regulated by the HC diet in *Wt* and *Lrp5*^*−/−*^ mice, although in a non-significant manner (Fig.6B). Finally, *Lrp6* mRNA expression levels were increased by 31 ± 2% in HC *Lrp5*^*−/−*^ mice with respect to NC *Lrp5*^*−/−*^ mice (Fig.6C). Regression analyses revealed a significant positive correlation between *Lrp6* gene expression levels and lipid rich coverage in aortas of *Lrp5*^*−/−*^ mice, but not for *Lrp1* (Fig.6D).

## Discussion

Over the last decades, LRP5 has been involved in several pathways, including bone development, impaired fat tolerance and glucose metabolism 8,17–19. Impaired plasma clearance of chylomicron remnants and reduced glucose tolerance was observed in LRP5-deficient mice fed a high-fat diet compared with their *Wt* littermates 17. Here, we have studied the role of LRP5 and the canonical Wnt pathway in mildly HC *Lrp5*^*−/−*^ mice. Total cholesterol and non-HDL plasma levels increased after HC feeding in *Wt* and *Lrp5*^*−/−*^ mice. However, total cholesterol levels in HC *Lrp5*^*−/−*^ mice that had a significantly higher aortic lipid infiltration were higher than those in HC *Wt* mice, suggesting an involvement for LRP5 during dyslipidaemia, at the initial stages of atherosclerosis development. Similarly in humans, LPR5-rs3736228T alleles that cause loss-of-function in LRP5 protein show increased plasmatic cholesterol levels in Chinese Han population 20 and is considered an independent risk factor for hypercholesterolaemia in the male Japanese population 21.

Hypercholesterolaemic *Lrp5*^*−/−*^ mice show larger lipid infiltration in the thoracic aorta compared with HC *Wt* animals. Accordingly, Immunohistochemistry analyses revealed an increase in macrophage staining in the intima layer of HC *Lrp5*^*−/−*^ mice aortas confirming our previous *in vitro* results showing that monocytes that overexpress LRP5 show a down-regulation of the differentiation processes by the sequestration of β-CATENIN to the cell membrane 12. Consequently, here we show that mice lacking LRP5 have more macrophages in the vascular wall. Activated monocytes/macrophages stimulate cytokine secretion promoting pro-inflammatory chronic stimulation 1,22,23. Therefore, we analysed the expression of pro-inflammatory and pro-atherogenic cytokines (*Il18*, *Il15* and *Il1*β) in white blood cells from HC *Wt* and HC *Lrp5*^*−/−*^ mice 24–27. Wnt/β-CATENIN activation inhibits the inflammatory response in endothelial cells 9 and enhances injury repair and healing responses in other inflammatory diseases including rheumatoid arthritis and colitis 28. We found increased inflammatory cytokines gene expression in white blood cells from HC *Lrp5*^*−/−*^ mice indicating increased inflammation.

IHC analyses revealed a down-regulation of the Wnt canonical pathway members, β-CATENIN and MMP7, in the absence of LRP5 evidencing a down-regulation of the canonical Wnt signalling pathway in HC *Lrp5*^*−/−*^ mice aortas. This supports our previous *in vitro* findings showing that LRP5 silencing in human macrophages abrogates Wnt/β-CATENIN pathway activation 13.

Aortic *Lrp6* expression increased in *Lrp5*^***−**/**−***^ animals fed the HC diet and directly correlated with aortic lesion area. LRP6 has been found overexpressed in human atherosclerotic lesions 29 and regulates LDLR-mediated LDL uptake as LDLR internalization is severely diminished in *Lrp6*^***−**/**−***^ cells 30. We analysed the expression of other receptors that have been described to participate in lipid internalization. Indeed, LRP2 contributes to HDL metabolism by internalizing ApoA-I and ApoA-II, which are structural components of HDLs 31; LRP8 is a component of the interactions between the endothelium and monocytes and leucocyte transendothelial migration, foam cell formation and activation of platelet aggregation 32; and the classical LDLR is known for its involvement in lipoprotein transport and plasmatic LDL cholesterol clearance 33. *Lrp2*, *Lrp8* and *Ldlr* expression levels were down-regulated in aortas from HC *Lrp5*^***−**/**−***^ indicating that they were not contributing to the observed lipid deposition in mice aortas. The atherogenicity of CD36 remains unclear, CD36 deficiency has been associated with enhanced atherosclerotic cardiovascular diseases 34, but cultured macrophages from these patients present a reduced uptake of oxidized LDL 35. Our results do not support the contribution of *Cd36* to the lipid-rich phenotype of *Lrp5*^***−**/**−***^ mice aortas as its expression is increased in HC *Wt* mice but reduced in HC *Lrp5*^***−**/**−***^ mice. *Lrp1* is up-regulated in aortas from *Lrp5*^***−**/**−***^ mice. LRP1 has been found up-regulated in advanced human atherosclerotic plaques where its up-regulation is induced by extracellular lipids in human smooth muscle cells and human macrophages 36,37. Up-regulated LRP1 expression is also found in the aorta of rabbits and pigs after HC diets suggesting a pro-atherogenic role for LRP1 overexpression 36,38. Consistently, here we show enhanced *Lrp1* expression levels in HC conditions in *Wt* and *Lrp5*^***−**/**−***^ mice. *Vldlr*, a multiligand receptor that binds VLDL and chylomicron remnants 39 is not highly modified by mild hypercholesterolaemia or LRP5^***−***/***−***^.

Interestingly, there was an increase in *Lrp6* gene expression levels in aortas of *Lrp5*^***−**/**−***^ animals after HC feeding; however, *Lrp6* did not trigger the canonical Wnt pathway. *Lrp5*^***−**/**−***^ and *Lrp6*^***−**/**−***^ mice have different phenotypes suggesting that these receptors cannot compensate each other's function 40–42. Also, mammary stem cells require LRP5 to trigger Wnt signalling despite of LRP6 co-expression 42. In view of our results, we suggest that *Lrp1* and *Lrp6* contribute to the increased aortic lipid deposition observed in HC *Lrp5*^***−**/**−***^ mice, but further analysis on the LRP1-LRP5 and LRP6-LRP5 interactions are needed to better characterize this hypothesis.

In conclusion, our findings show that lipid depositions in the aorta are larger in HC *Lrp5*^*−/−*^ mice with mildly increased levels of blood cholesterol demonstrating a protector role for LRP5 in the vascular wall. LRP5 and proteins from the Wnt/β-CATENIN pathway are up-regulated in HC *Wt* aortas and this increase is lost when LRP5 is absent. The absence of LRP5 regulates the dyslipidaemic profile by promoting lipid and macrophage retention in the vessel wall and increasing leucocyte driven systemic inflammation.
